# Guinea worm infection in northern Nigeria: reflections on a disease approaching eradication

**DOI:** 10.1111/tmi.12855

**Published:** 2017-04-05

**Authors:** Brian Greenwood, Alice Greenwood, Andrew Bradley

**Affiliations:** ^1^ Faculty of Infectious and Tropical DiseasesLondon School of Hygiene & Tropical MedicineLondonUK; ^2^ Malumfashi Endemic Diseases Research ProjectAhmadu Bello UniversityZariaNigeria

**Keywords:** guinea worm, morbidity, immunology, eradication, vers de Guinée, morbidité, immunologie, éradication

## Abstract

Global eradication of the guinea worm (*Dracunculus medinensis*) is near, although perhaps delayed a little by the discovery of a transmission cycle in dogs. It is therefore an appropriate time to reflect on the severe impact of this infection on the life of the communities where it was endemic prior to the start of the global eradication programme in 1981. From 1971 to 1974, we conducted a series of unpublished studies on guinea worm in a group of villages in Katsina State, northern Nigeria, where the infection was highly endemic. These studies demonstrated the high rate of infection in affected communities, the frequent recurrence of the infection in some subjects and the long‐standing disability that remained in some infected individuals. Immunological studies showed a high level of immediate hypersensitivity to adult worm and larval antigens but a downregulation of Th1‐type T‐cell responses to worm antigens. Freeing communities such as those described in this article from the scourge of guinea worm infection for good will be an important public health triumph.

## Abstract (fr)

L’éradication mondiale du ver de Guinée (*Dracunculus medinensis*) est proche, quoique peut‐être un peu retardée par la découverte d'un cycle de transmission chez les chiens. Il est donc opportun de réfléchir à l'impact sévère de cette infection sur la vie des communautés où elle était endémique avant le début du programme mondial d’éradication en 1981. De 1971 à 1974, nous avions mené une série d’études non publiées sur le ver de Guinée dans un groupe de villages dans l'Etat de Katsina, dans le nord du Nigeria, où l'infection était fortement endémique. Ces études ont démontré le taux élevé d'infection dans les communautés affectées, la récurrence fréquente de l'infection chez certains sujets et le handicap de longue durée qui restait chez certaines personnes infectées. Des études immunologiques ont montré un niveau élevé d'hypersensibilité immédiate aux vers adultes et aux antigènes larvaires mais une régulation négative des réponses des lymphocytes T de type TH1 aux antigènes du ver. Libérer pour de bon les communautés telles que celles décrites dans ce document du fléau de l'infection par le ver de Guinée sera un triomphe important de la santé publique.

## Abstract (es)

La erradicación global del gusano de Guinea (*Dracunculus medinensis*) está cerca, aunque tal vez se ha retrasado un poco por el descubrimiento de un ciclo de transmisión en perros. Por lo tanto, es el momento apropiado de reflexionar sobre el impacto severo que esta infección tiene sobre la vida de las comunidades en donde era endémica antes del inicio del programa global de erradicación en 1981. Entre 1971‐74 realizamos una serie de estudios no publicados sobre el gusano de Guinea en un grupo de poblados del Estado de Katsina, al norte de Nigeria, en donde la infección era altamente endémica. Estos estudios demostraron la alta tasa de infección en las comunidades afectadas, la frecuente recurrencia de la infección en algunos sujetos y la discapacidad prolongada en algunos de los individuos infectados. Los estudios inmunológicos mostraban un alto nivel de hipersensibilidad a los antígenos del gusano adulto y de las larvas pero una regulación decreciente de la respuesta de inmunidad celular TH1 frente a los antígenos del gusano. Liberar definitivamente a comunidades como las descritas en este artículo del flagelo del gusano de Guinea, será un importante triunfo para la salud pública.

## Introduction

Thanks to the work of The Carter Foundation, WHO, other international organisations and local communities, eradication of the guinea worm *Dracunculus medinensis* is imminent 1, 2, although perhaps delayed a little by the discovery of a transmission cycle in dogs in Chad 3, 4, 5. The importance of this achievement should not be underestimated for the devastating impact of this infection, when uncontrolled, has frequently been neglected. In this article, we describe our experience of this infection in northern Nigeria 40 years ago, when it was a truly neglected disease, to emphasise the public health importance of its imminent eradication.

In the early 1970s, when the studies described in this article were carried out, guinea worm was almost completely unrecognised as a significant health problem in Nigeria and elsewhere as severely affected subjects were usually too disabled to travel to the nearest clinic and they knew that there was little that conventional medicine could offer them. Thus, at the request of an anthropologist living in a severely affected community in northern Nigerian, we initiated a series of studies to describe the clinical features of guinea worm infections, its impact on the community and why patients infected with a large worm accessible to the immune system did not develop a protective immune response. None of the findings from these studies has been published due to lack of interest from the scientific and public health communities at the time that they were carried out. However, as the day approaches when the world can celebrate the eradication of this infection, we believe that it is worth recording how guinea worm affected a group of Nigerian villages prior to the initiation of the eradication programme in 1981.

## Methods

### Study area

**Figure 1:**
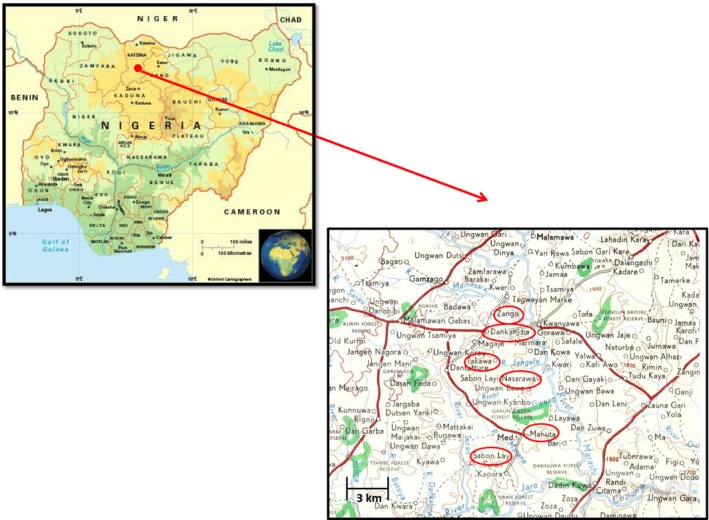
Map of Nigeria showing the site of the study in Katsina State, Nigeria, and a more detailed map of the study area indicating the area covered by the Malumfashi Endemic Diseases Research Project. Villages circled in red had more than 10 cases.

**Figure 2:**
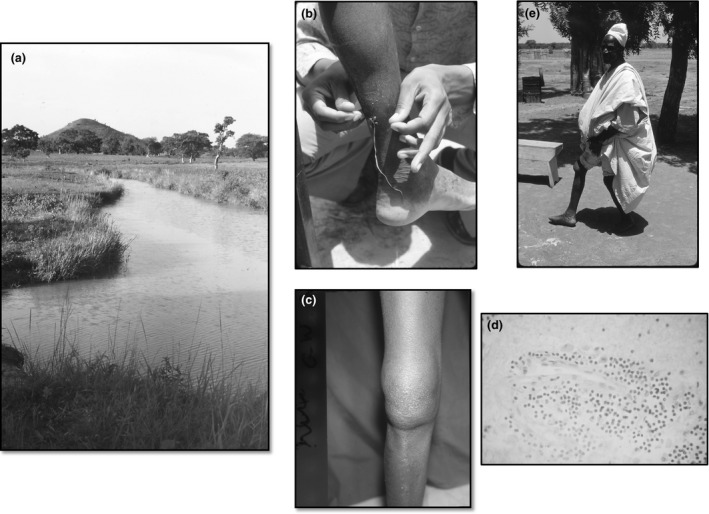
(a) Typical pool used for washing and as a source of drinking water during the farming season, (b) local extraction of a guinea worm using a twig, (c) arthritis of the knee in a patient with a guinea worm emerging above the medial malleolus, (d) synovial needle biopsy from the patient shown in panel c showing infiltration of the synovium with mononuclear cells, (e) long‐standing damage to the knee and permanent disability following guinea worm arthritis of the knee.

The study was conducted in a group of villages near to the town of Malumfashi, northern Nigeria, which were part of the Malumfashi Endemic Diseases Research Project established through a partnership between the Institute of Health, Ahmadu Bello University (ABU) and the Liverpool School of Tropical Medicine (Figure 1)**.** The ecological and demographic characteristics of the area have been reported previously 6. Most of the villages in the study area had a hand‐pump well and additional private wells within a household were usually in a good state of repair. However, during the rainy season, small pools that formed between villages were used for washing and as a source of drinking water during farming (Figure 2a). Many of these pools harboured *Cyclops,* the intermediate host of the guinea worm.

### Household survey

After preliminary visits to the affected communities, the village of Dankanjiba was chosen as a typical village for the conduct of a retrospective survey. The village was mapped and a census obtained. After consultations with the village committee, all adult males were invited to participate in a survey that enquired about their previous experience of guinea worm infection and how this had affected their lives.

### Weekly clinics

Following this initial survey, weekly clinics were held in the study area throughout the rainy season for a period of 4 years (1971–1974), with visits alternating between villages each week. If a relative reported a case who was unable to walk to the clinic, the patient was visited at home. A history was taken from any subject who presented with a guinea worm and an examination performed. In 1974, a swab was collected from the site of an emerging worm for microbiological studies if this appeared infected in a consecutive series of 42 patients. Treatment with antibiotics was provided if there were signs of bacterial infection. Patients were requested to return for follow‐up at the next clinic so that the course of their illness could be followed but no formal follow‐up procedure was followed. In 1972, a small, open‐label trial of metronidazole was undertaken in 37 patients.

### Microbiology

Smears from ulcer sites were made and stained with Gram stain. Swabs were plated on blood agar plates within 6 h of collection and cultured aerobically and anaerobically for 48 h. The predominant colony was characterised by morphology and by Gram stain and the species identified using conventional microbiological techniques. Antibiotic sensitivity against penicillin, streptomycin, chloramphenicol, tetracycline and neomycin was determined using a disc method.

### Immunology

The immunological methods employed in the studies are described only briefly as these are now largely of historical interest.

#### Preparation of larval and adult worm antigens

Larvae were obtained by irrigating the head of a recently emerged worm with normal saline. The suspension was then subjected to ultrasound and concentrated by dialysis against Lyphogel. Segments of adult worm were obtained from subjects who were being treated locally by extraction of the worm. Segments of worm were cleaned, homogenised, heat‐inactivated at 56 °C for 10 min and filtered through a Millipore filter and the protein content of the extract determined. Adult and larval extracts were fractionated on a Sephadex G150 column.

#### Skin tests

Test subjects were injected intradermally with larval or adult antigen preparations at optimum concentrations, determined in initial experiments in expatriate volunteers who had not been exposed to guinea worm, and the size of the wheal present 10–15 min after injection was measured in two directions with the average recorded. Injection sites were examined 48–72 h after injection.

#### IgE assays

IgE concentrations were measured using a commercial radioimmunoassay (Behringwerke).

#### Precipitin reactions

Sera were tested for precipitating antibodies using the Ouchterlony technique, by which an optimum concentration of larval or worm antigen was placed in the central well of an agar plate and test and control sera in the surrounding wells. Plates were examined for precipitin lines 48–72 h later.

#### Haemagglutination assay

Tanned sheep red blood cells were coated with larval or adult antigen fractions at optimum concentrations determined in preliminary experiments. Test sera were absorbed overnight with uncoated red cells to absorb heterophile antibodies, and tested in doubling dilutions against the coated red cells. The highest dilution causing visible haemagglutination was recorded.

#### Cell mediated assays

Lymphocytes were obtained from infected and control subjects after separation on Lymphoprep and tested for proliferation after stimulation with larval or adult antigen at optimum concentrations using H^3^ thymidine incorporation after 72 h of culture.

### Analysis

Data obtained in the field were transcribed onto Excel spreadsheets and verified. Comparison of categorical variables was made using a chi‐square test, and Student's *t*‐test was used to compare means. For some immunological variables, variation by sex, age and number of previous guinea worm infections was determined.

### Ethical approval

The study was approved by the Director of Health Services, Ahmadu Bello University; the Paramount Chief for the study area (the Galadima of Malumfashi); and by village heads and their advisors. Oral, informed consent was obtained from study subjects for the collection of blood samples or for participation in skin testing.

## Results

### Retrospective survey of guinea worm infection

The population of Dankanjiba was estimated to be approximately 1200 in 1971 with 250 men over the age of 16 years; 213 (85%) of the latter participated in the initial survey. 72% of the interviewees had a clear recollection of infection with a guinea worm at some stage of their life. Many had had repeated infections with 21% reporting five or more episodes. The number of episodes recalled by age group is shown in Table 1. The mean number increased from 1.93 [95% CI: 1.07; 2.79] in 20‐ to 29‐year‐olds to 3.94 [95% CI: 2.30; 5.58] in the 40‐ to 49‐year‐old group but then fell to a mean of 2.27 [95% CI: 1.34; 4.20] in those aged 60 years or more. Approximately 10% of the adult male inhabitants had a guinea worm infection each year. Eleven interviewees (5.9%) reported that they had been unable to work for 3 months or more because of a guinea worm infection and three had been disabled for over a year. One subject reported that his wife had died from an acute illness associated with an episode of multiple guinea worm infections. There was no evidence of clustering in the village with infections having occurred in nearly all compounds.

**Table 1:** Mean numbers of previous guinea worm infections and resulting disability for 3 months or more in 185^a^ adult male inhabitants of Dankanjiba village, northern Nigeria, (1971). Range shown in brackets

Age in years	Number	Mean number of infections [95% CI] (range)	Number (%) with disability > 3 months
20–29	28	1.93 [1.07; 2.79] (0–5)	2 (7.1%)
30–39	67	3.06 [2.37; 3.75] (0–15)	4 (6.0%)
40–49	33	3.94 [2.30; 5.58] (0–15)	3 (9.1%)
50–59	35	3.49 [2.15; 4.83] (0–20)	2 (5.7%)
60 or >	22	2.27 [1.34; 4.20] (0–12)	0
Total	185	3.05 [2.54; 3.56] (0–20]	11 (5.9%)

a Thirty‐two respondents were unsure of whether or not they had had a previous infection.

### Clinical features of guinea worm infection

**Figure 3:**
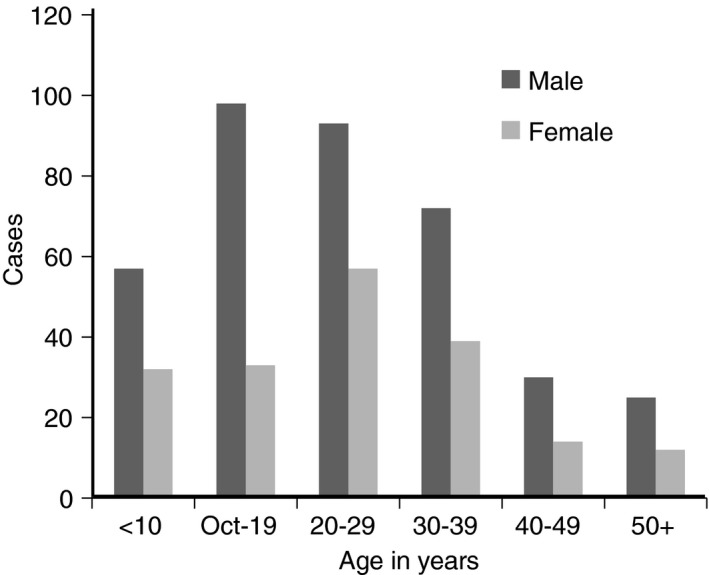
Age and sex distribution of 563 Nigerian cases of guinea worm infection.

A total of 563 patients with a patent guinea worm infection were seen during the rainy seasons of 1971–1974 (Table 2). Cases came from 47 villages scattered across the study area but they were concentrated in a few larger villages (Sabon Layi Suran: 208 cases; Dankanjiba: 96 cases; and Mahuta: 62 cases) (Fig. 1). Two‐thirds of the subjects were male. The mean age of affected male subjects (25.8 years [95% CI: 23.9; 27.7]) and female subjects (26.9 years [95% CI: 23.7; 30.1]) was similar (Figure 3). Cases reported a mean of 1.47 [95% CI: 1.21; 1,75] previous episodes of guinea worm infection; 38 subjects (6.71%) reported having been infected on five or more occasions. The average duration of symptoms prior to presentation was 2.61 weeks [95% CI: 2.34; 2.88] (Table 2). One thousand and twenty worms were seen, the majority (88.4%) of which were emerging from a leg (Figure 2b). Approximately one‐half of cases had signs of secondary infection at the site of worm emergence requiring treatment with an antibiotic (Table 2). The average duration of active infection was 5.94 [95% CI: 5.93; 6.37] weeks but some subjects still had symptoms at that time and were not followed actively until asymptomatic. A minority of cases were disabled for a much longer period of time with at least 98 cases (17.4%) having an active infection which persisted for 10 weeks or more.

**Table 2:** Summary of the findings in 563 Nigerian patients with a patent guinea worm infection

Variable	Year seen				
	1971	1972	1973	1974	Total
Number of cases	178	113	152	122	563
Sex					
Male	135	83	88	73	380
Female	43	30	64	49	183
Age in years (mean [95% CI])					
Male	26.4 [23.9, 28.9]	24.5 [21.3, 27.7]	23.8 [20.6, 27.0]	25.1 [21.7, 28.5]	25.8 [23.9, 27.7]
Female	27.1 [22.6, 31.6]	24.5 [18.4, 30.6]	25.5 [22.0, 29.0]	24.8 [21.1, 28.5]	26.9 [23.7, 30.1]
Number of previous infections					
Mean [95% CI]	1.9 [1.3, 2.4]	1.3 [1.3, 2.4]	1.1 [0.8, 1.9]	1.5 [0.9, 2.0]	1.5 [1.2, 1.8]
Duration of symptoms (weeks)^a^					
Mean [95% CI]	2.7 [2.3, 3.6]	2.5 [2.1, 2.9]	3.0 [2.2, 3.8]	2.0 [1.7, 2.3]	2.6 [2.3, 2.9]
Duration of illness (weeks)^b^					
Mean [95% CI]	6.5 [5.7, 7.3]	4.2 [3.6, 4.8]	7.2 [6.2, 8.2]	5.0 [4.5, 5.6]	5.9 [5.5, 6.3]
Worms present per person^a^					
1	23	65	92	79	359
2–4	47	35	55	38	175
5 or more	6	13	5	5	29
Site of worm emergence					
Leg	283	190	241	188	902
Arm	15	13	17	21	66
Torso	15	11	9	11	46
Other	5	0	1	0	6
Infection at site of emergence					
Number (%)	131 (41.5%)	88 (41.1%)	129 (48.1%)	77 (35.0%)	425 (41.7%)
Definite arthritis					
Number (%)	13 (7.3%)	5 (4.4%)	6 (3.9%)	2 (1.6%)	26 (4.6%)
Possible arthritis					
Number (%)	31 (17.4%)	8 (7.1%)	30 (19.7%)	12 (9.8%)	81 (14.4%)
Site of definite arthritis					
Knee	7	2	5	2	16
Ankle	5	1	1	0	7
Wrist	0	2	0	0	2
Shoulder	1	0	0	0	1

a Data missing for 10 subjects.

b Data missing from six subjects.

#### Arthritis

Twenty‐six cases of arthritis, defined as a tender, swollen joint with limitation of movement, were detected during weekly clinics. The mean age of these patients was 24.72 [95% CI: 19.34; 30.10] years and the majority (19) were male. Their mean duration of symptoms before presentation was 2.63 [95% CI: 1.56; 3.70] weeks. Most patients had an obvious guinea worm ulcer near to the affected joint but in two cases the arthritis appeared before the guinea worm. The knee was the most frequently affected joint (Figure 2c) but arthritis of the ankle, wrist and shoulder was also seen (Table 2). An additional subject was seen with an ankylosed right knee, fixed at 45%, causing severe difficulty in walking, which had resulted from a guinea worm infection a year previously (Figure 2e). Another nine patients with guinea worm arthritis were seen at a health centre in the area or at the local hospital, and synovial aspiration was performed in these subjects. Two patterns were seen – in six subjects, viscous fluid was obtained with a relatively low white blood cell count (300, 600, 800, 2000, 7000 and 17 000, cells per μl), a predominance of lymphocytes and monocytes and a sterile culture. A synovial needle biopsy obtained from one of these patients with a large knee effusion showed infiltration of the synovium with mononuclear cells (Figure 2d). Purulent synovial fluid was obtained from the remaining three patients with a high white blood cell count (27 000, 200 000, 300 000 cells per μl) and a predominance of polymorphonuclear neutrophil leucocytes. *Staphylococcus aureus* was obtained from two of these cases and an *α*‐haemolytic streptococcus from the third. All the patients made a full recovery.

#### Microbiological findings

Swabs were obtained from ulcers from 42 consecutive subjects over a two‐month period in 1974. *S. aureus* was the most frequently isolated bacterium (19 isolates) followed by *β*‐haemolytic streptococci (seven isolates) and *α*‐haemolytic streptococci (seven isolates). There was one isolate of *Escherichia coli* and one of a *Klebsiella* species. Spore‐bearing gram‐positive rods were cultured on three occasions and seen in direct smears from the ulcer in a further two subjects. Eleven of the 15 *S. aureus* isolates tested were penicillin‐resistant.

#### Treatment

Patients with an active infection were treated with analgesics and a simple dressing if an ulcer was present and discouraged from exposing the lesion to water. Penicillin or chloramphenicol was given if there was evidence of secondary bacterial infection or purulent arthritis. Following reports of the potential benefit of metronidazole 7, 8, 37 patients were allocated randomly to receive aspirin or aspirin plus metronidazole in a dose of 400 mg/day for 7 days and followed weekly for 4 weeks using a severity score (maximum 20). This declined from 7.65 [95% CI: 5.37; 10.23] to 3.57 [95% CI: 1.95; 5.19] in the aspirin alone group and from 8.00 [95% CI: 6.33; 9.67] to 4.10 [95% CI: 2.61; 5.59] in the aspirin plus metronidazole group, an almost identical rate of recovery (*P* > 0.5).

### Immunology

#### IgE concentrations

Serum total IgE levels were measured in 30 randomly selected adult subjects with an active guinea worm infection and compared with the values seen in 20 adult blood donors who attended Ahmadu Bello University Hospital, Zaria. The mean value obtained in the subjects with an active guinea worm infection was significantly higher than the mean value found in healthy blood donors (12 246 IU/ml [95% CI: 10 380; 13 610] and 6607 IU/ml [95% CI: 5720; 7493] respectively) (*P* = 0001).

#### Immediate hypersensitivity skin tests

Preliminary tests with varying concentrations of adult and larval antigen preparations in healthy adult, expatriate volunteers showed that concentrations of approximately 5 μg/ml protein did not produce any immediate reactivity. Using this antigen concentration, tests were conducted in 51 randomly selected subjects with an active guinea worm infection and in 19 control subjects who stated that they had never had a guinea worm infection. The mean increase in size of the bleb at the site of intradermal injection of adult antigen 15 min after injection was greater in the guinea worm patients than in the controls (8.10 [95% CI: 6.82; 9.28] mm compared with an increase of 5.86 [95% CI: 3.87; 7.85] mm in the controls) (*P* = 0.04). A similar result was found for larval antigen with increases of 8.54 [95% 7.17; 9.91] mm and 4.54 [95% CI: 2.78; 6.30] mm being recorded in cases and controls respectively (*P* < 0.001). No correlation was found between age or severity of infection and the immediate response. However, for adult worm antigen, 25 subjects with a history of prior infections above the median number had a smaller reaction than did 24 subjects with a history of less frequent past infections (mean 1.20 [95% CI: 0.61; 1.79] *vs*. 2.87 [95% CI: 1.46; 4.28] [*P* = 0.03). A similar pattern was seen for larval antigen, although the difference between groups was not statistically significant (1.23 [95% CI: 0.43; 2.03] *vs*. 2.90 [0.81; 4.99] [*P* = 0.13]).

#### Precipitin reactions

Precipitin lines were detected significantly more frequently in a randomly selected group of guinea worm infected subjects than in northern Nigerian blood donors who did not have a history of guinea worm infection (22/96 [23%] *vs*. 2/50 [4%]) (*P* = 0.003) (Table 3). Similar results were obtained for the larval antigen, which was detected in 15/97 [15%] infected subjects but in only 1/50 [2%] controls (*P* < 0.001). No correlation with sex was noted. Subjects who were positive against adult antigen tended to be younger than those who were negative (mean age 20.45 [95% CI: 15.33; 25.57] years *vs*. 27.72 [95% CI: 25.02; 30.42]) years (*P* = 0.014) but the opposite trend was seen for larval antigen with the mean age of positive subjects being 31.80 [95% CI: 23.11; 40.49] years *vs*. 25.62 [95% CI: 23.22; 28.02] years in those who were negative (*P* = 0.07), so this may have been a chance finding. The number of previous infections reported by positive reactors compared with those who were negative was similar for both adult antigen (1.54 [95% CI: 0.66; 2.42] *vs*. 1.78 [95% CI: 1.18; 2.63]) and for larval antigen (1.60 [95% CI: 0.39; 2.83] *vs*. 1.73 [95% CI: 1.19; 2.50]) (*P* > 0.5 for each comparison). Six sera showing strong positive reactions were tested against different fractions of adult extract obtained on Sephadex G150 fractionation. Bands were seen most frequently with fractions of molecular weight in the range of 20 000–100 000.

**Table 3:** Serological findings in patients with active guinea worm and in healthy adult Nigerian blood donors

	Guinea worm cases	Blood donors
Precipitating antibody		
Adult worm antigen		
Number tested	96	50
Number of precipitin lines (%)		
0	74 (77.0%)	48 (96.0%)
1	14 (14.6%)	2 (4.0%)
2	6 (6.3%)	0
3 or >	2 (2.1%)	0
Precipitating antibody		
Larval antigen		
Number tested	97	20^a^
Number of precipitin lines (%)		
0	75 (77.3%)	19 (95.0%)
1	14 (14.4%)	1 (5.0%)
2	6 (6.2%)	0
3 or >	2 (2.1%)	0
Haemagglutinating antibody		
Adult antigen		
Number tested	101	50
Number positive (%)	99 (98.0%)	18 (36.0%)
Mean titre of positives		
(log_2_) [95% CI]	7.88 [7.23; 8.45]	2.17 [1.74; 2.60]

a Only 20 samples were tested because of shortage of antigen.

#### Haemagglutinating antibodies

Haemagglutinating antibodies to adult antigen at a titre of 1:4 or > were found in nearly all subjects with guinea worm (99/101) (99%), compared with a prevalence of 18/50 (36%) in adult male northern Nigerian blood donors (Table 3). The mean titre (log_2_) of the guinea worm patients who were positive (7.84 [95% CI: 7.23; 8.45] was much higher than that found in the blood donors (2.17 [95% CI: 1.74; 2.60) (*P* < 0.001). Those in the age group ≥50 years nearly all had very high titres (mean 8.50 [95% CI: 6.98; 10.02]. A significant association was noted with the number of previous episodes reported with mean titres being 6.58 [95% CI: 5.22; 7.94] in those who reported never having been infected previously, 7.62 [95% CI: 6.89; 8.35] in those with a history of 1–4 previous infections and 9.54 [95% CI: 7.34; 11.74] in those with a history of five or more infections (*P* = 0.03). Haemagglutinins against red blood cells coated with larval antigen at a titre of 1:4 or greater were found in only 1/25 guinea worm infected subjects and in none of 20 blood donor controls.

#### Cell mediated immune responses

Skin tests with adult worm antigen were examined for induration 48 h after injection in 50 subjects with an active guinea worm infection. Only three reactions (6%) >5 mm in diameter were observed. In contrast 75% (21/28) had a positive reaction of ≥5 mm diameter after intradermal challenge with 10 TU of tuberculin. Lymphocytes from 10 male patients aged 14–35 years with active guinea worm were cultured with varying concentrations of adult or larval antigen. No subject had a rate of proliferation more than twice that of the control culture at any of the antigen concentrations tested.

## Discussion

Weekly visits during the rainy seasons of 1971–1974 to a group of villages typical of many across northern Nigeria emphasised the debilitating effects of guinea worm on the community with a significant proportion of the adult male population being disabled during the peak farming season. It is likely that this had an adverse effect on the nutrition and health of their families during the coming year, as found in several subsequent studies 9, 10, 11, 12, 13, 14. Guinea worm infection was also an important cause of absence from school, as noted elsewhere in Nigeria 15.

Guinea worm ulcers readily become secondarily infected unless carefully managed and this was the case in this study with many subjects developing a surrounding cellulitis. The most frequently isolated bacteria from infected ulcers were *S. aureus* and haemolytic streptococci. Tetanus is a well‐recognised complication of guinea worm infection after contamination of the wound from soil. No cases of tetanus were observed in this study, although one interviewee reported that his wife had died from an acute febrile illness associated with infection by several guinea worms and it is possible that this was a case of tetanus. Gram‐positive, spore‐bearing rods were grown from two subjects although these were not differentiated further.

Spread of infection to an adjacent joint can lead to a purulent arthritis, which, if not managed well, can result in long‐term deformity 16, 17. Purulent arthritis was seen in three patients in this series. In other patients with arthritis, examination of synovial fluid was sterile and showed a relatively low white blood cell count characterised by a predominance of lymphocytes and monocytes, as seen in a previous study in southern Nigeria 18 suggesting that the arthritis resulted from an immunological reaction to an adjacent worm. Reports that treatment with metronidazole accelerated recovery 7, 8 were not confirmed, as was the case when this drug was tried in Ghana 19.

The severe reaction that may follow rupture of an adult worm during extraction has long been recognised and attributed to an allergic reaction. This supposition is supported by the observations that nearly all infected subjects showed an immediate hypersensitivity to adult and larval antigens, a reaction first described in patients seen in northern Nigeria in 1935 20, and that infected subjects’ had a higher overall serum IgE level than blood donors from the same area. Similar results were found in a subsequent study carried out in Ilorin, Nigeria, with infected subjects having a high incidence of positive immediate skin tests and higher IgE concentrations than controls 21. However, higher total IgE levels were not found in a subsequent study in Ghana 22. High haemagglutinin antibody titres to worm antigen were found in nearly all infected subjects and were highest in those with a history of several prior infections; precipitating antibodies were detected less frequently. Bloch *et al*. 23 have subsequently investigated the antibody response to guinea worm infection in more detail. Antibodies of the IgG4 subclass gave the best discrimination between cases and controls resident in an area that was not endemic for guinea worm.

Little evidence was found that infected subjects developed any protective immunity. The infection rate was lower in older than middle‐aged men but this may have been due to less exposure of the older men. Many subjects had recurrent infections each year for many years. It seems likely that the guinea worm has the ability to downregulate aspects of the immune response that might contribute to protection and a clue as to what this might be was found by the inability to detect any evidence of a cellular immune response to adult antigen by delayed hypersensitivity skin testing or by lymphocyte proliferative studies. Aiyedun *et al*. 21 also noted an absence of a delayed skin test response in guinea worm patients, and Knoop *et al*. 24 subsequently showed a diminished ThI‐type 1 cytokine response in subjects with an active infection together with enhanced IL‐10 production on stimulation with guinea worm and cross‐reactive antigens in keeping with these earlier findings.

The results presented in this study have a number of weaknesses. The patient population was relatively unselected and some very severe cases may have been missed despite home visits being made when requested. Selection of subjects for individual substudies was not performed as rigorously as would now be required and for the immunological studies the control group was locally recruited blood donors rather than age, sex and community‐matched controls as would have been preferable. Nevertheless, it is likely that the main conclusions from the immunological studies are valid.

As the world prepares to celebrate the eradication of the guinea worm, it is important to remember the massive health and social burden caused by this worm in communities such as the ones described in this article and the magnitude of the achievement in ridding the world of this debilitating infection.
